# Supplementary material for: Environmental and Genetic Risk Factors in Developmental Dysplasia of the Hip for Early Detection of the Affected Population
Source: Diagnostics (Basel). 2024 Apr 25;14(9):898. doi: 10.3390/diagnostics14090898 (PMC11083091; doi:10.3390/diagnostics14090898)
Supplement: Supplementary file 1 [file diagnostics-14-00898-s001.zip › MDPI_SupplementaryFigureS1.pdf]

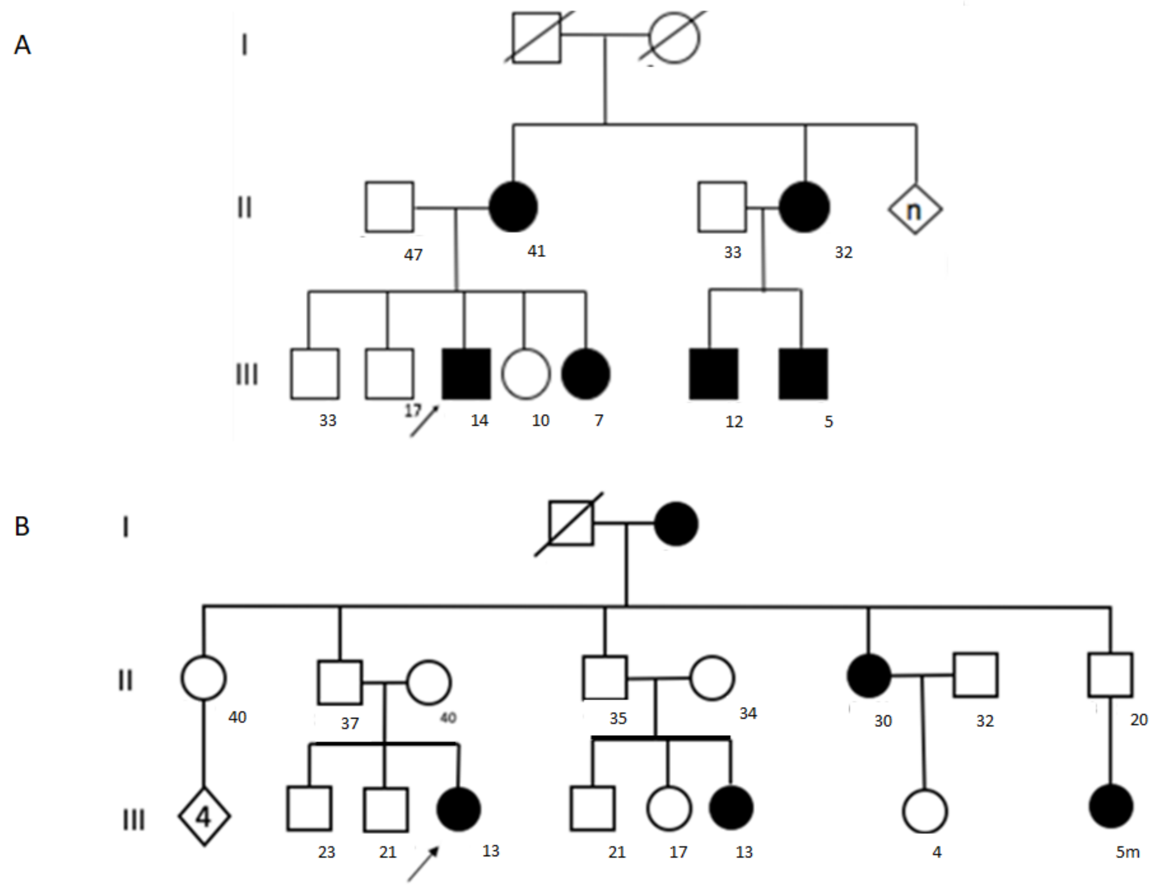

Supplementary Figure S1. Pedigrees showing DDH transmission. (**A**) Family resembling autosomal dominant inheritance. (**B**) Family with three affected generations, without a clear Mendelian pattern. Numbers represent age in years, except where the 'm' is shown, which represents months. Arrows represent individuals included in the study.
