# Supplementary material for: Environmental and Genetic Risk Factors in Developmental Dysplasia of the Hip for Early Detection of the Affected Population
Source: Diagnostics (Basel). 2024 Apr 25;14(9):898. doi: 10.3390/diagnostics14090898 (PMC11083091; doi:10.3390/diagnostics14090898)
Supplement: Supplementary file 1 [file diagnostics-14-00898-s001.zip › MDPI_SupplementaryFigureS2.pdf]

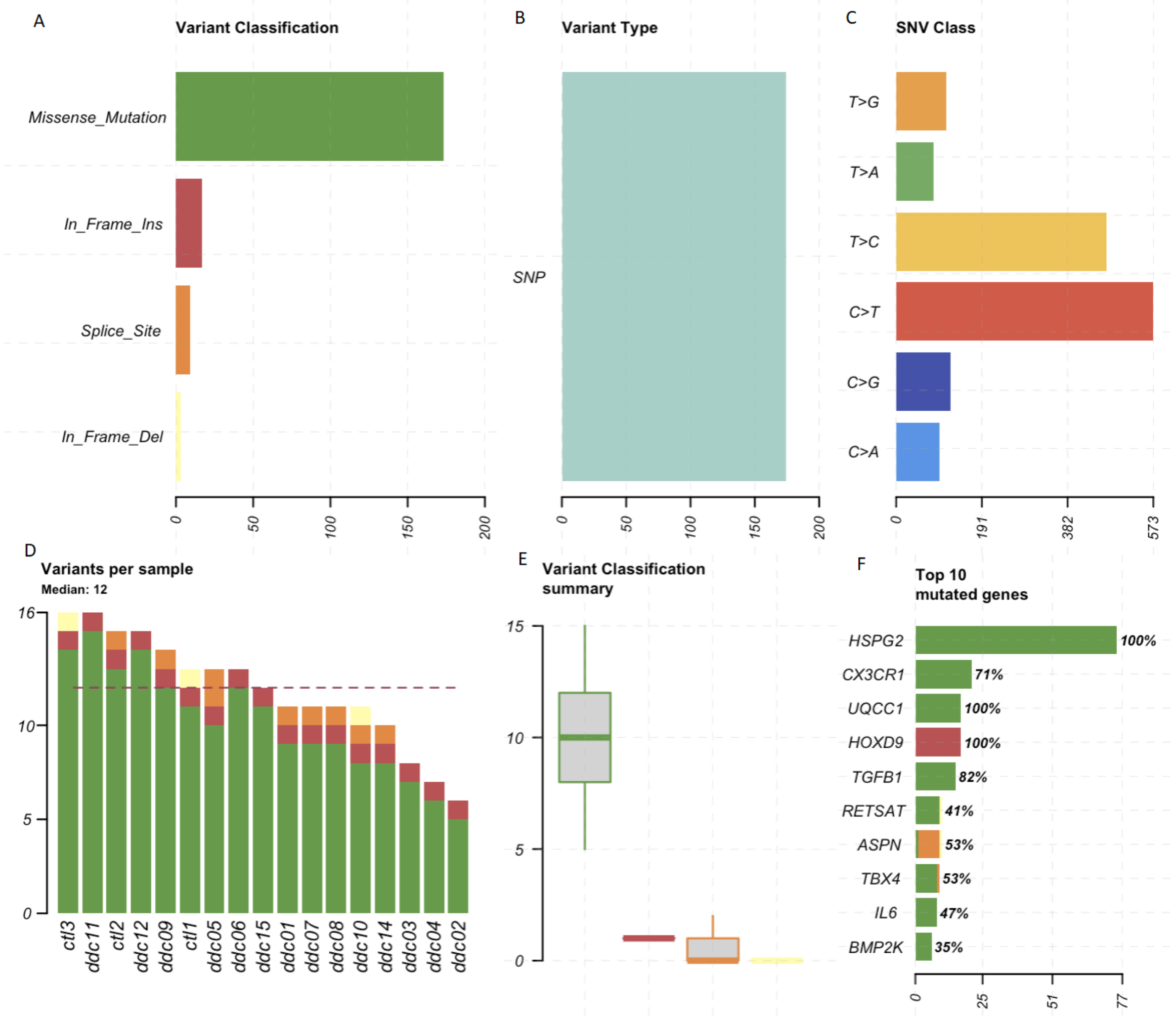

Supplementary Figure S2. SNV description. SNVs were classified based on the consequence at the RNA level (**A**) and type (**B**). SNVs class was also quantified, being C>T transition the most frequent (**C**). The median of variants per sample was 12 (**D**), missense variants were the most frequent (**E**). *HSPG2* presented the highest number of variants and variants were found in the 100% of the analyzed individuals (**F**). ctl corresponds to control. Ddc corresponds to the individuals with developmental dysplasia of the hip.
